# Chemometric Analysis of Urinary Volatile Organic Compounds to Monitor the Efficacy of Pitavastatin Treatments on Mammary Tumor Progression over Time

**DOI:** 10.3390/molecules27134277

**Published:** 2022-07-03

**Authors:** Paul Grocki, Mark Woollam, Luqi Wang, Shengzhi Liu, Maitri Kalra, Amanda P. Siegel, Bai-Yan Li, Hiroki Yokota, Mangilal Agarwal

**Affiliations:** 1 Department of Chemistry and Chemical Biology, Indiana University—Purdue University, Indianapolis, IN 46202, USA; pgrocki@iu.edu (P.G.); mwoollam@iu.edu (M.W.); apsiegel@iupui.edu (A.P.S.); 2 Integrated Nanosystems Development Institute, Indiana University—Purdue University, Indianapolis, IN 46202, USA; hyokota@iupui.edu; 3 Department of Pharmacology, School of Pharmacy, Harbin Medical University, Harbin 150081, China; luqicmu160@163.com (L.W.); szliu@ccmu.edu.cn (S.L.); liby@ems.hrbmu.edu.cn (B.-Y.L.); 4 Department of Biomedical Engineering, Indiana University—Purdue University, Indianapolis, IN 46202, USA; 5 Hematology and Oncology, Ball Memorial Hospital, Indiana University Health, Muncie, IN 47303, USA; mkalra@iuhealth.org; 6 Simon Comprehensive Cancer Center, Indiana University School of Medicine, Indianapolis, IN 46202, USA; 7 Indiana Center for Musculoskeletal Health, Indiana University School of Medicine, Indianapolis, IN 46202, USA; 8 Biomechanics and Biomaterials Research Center, Indiana University—Purdue University, Indianapolis, IN 46202, USA; 9 Department of Mechanical & Energy Engineering, Indiana University—Purdue University, Indianapolis, IN 46202, USA

**Keywords:** volatile organic compounds (VOCs), gas chromatography (GC), mass spectrometry (MS), solid-phase microextraction (SPME), breast cancer biomarkers, pitavastatin, principal component analysis (PCA), linear discriminant analysis (LDA)

## Abstract

Volatile organic compounds (VOCs) in urine are potential biomarkers of breast cancer. Previously, our group has investigated breast cancer through analysis of VOCs in mouse urine and identified a panel of VOCs with the ability to monitor tumor progression. However, an unanswered question is whether VOCs can be exploited similarly to monitor the efficacy of antitumor treatments over time. Herein, subsets of tumor-bearing mice were treated with pitavastatin at high (8 mg/kg) and low (4 mg/kg) concentrations, and urine was analyzed through solid-phase microextraction (SPME) coupled with gas chromatography-mass spectrometry (GC-MS). Previous investigations using X-ray and micro-CT analysis indicated pitavastatin administered at 8 mg/kg had a protective effect against mammary tumors, whereas 4 mg/kg treatments did not inhibit tumor-induced damage. VOCs from mice treated with pitavastatin were compared to the previously analyzed healthy controls and tumor-bearing mice using chemometric analyses, which revealed that mice treated with pitavastatin at high concentrations were significantly different than tumor-bearing untreated mice in the direction of healthy controls. Mice treated with low concentrations demonstrated significant differences relative to healthy controls and were reflective of tumor-bearing untreated mice. These results show that urinary VOCs can accurately and noninvasively predict the efficacy of pitavastatin treatments over time.

## 1. Introduction

Breast cancer is the most common cancer diagnosed in women worldwide, comprising ~30% of all diagnosed cancer cases in women in 2021 [1]. Although most diagnosed, breast cancer has high treatment potential, especially if detected in the earlier stages [2]. Current breast cancer screening methods include mammography, ultrasound, and MRI [3]. However, these methods have limitations with regard to diagnostic sensitivity/specificity [4]. Nonetheless, confirmatory diagnostics for breast cancer is undertaken through biopsies, and breast cancer staging is performed using pathological grading as well as imaging with ultrasound and CT/PET scans [5]. After breast cancer diagnostics, breast cancer treatment regimens are implemented. Stage 0 breast cancer (ductal carcinoma in situ, DCIS) is treated with surgery (mastectomy or breast-conserving surgery) with or without radiation therapy. However, although most cases of DCIS do not progress, there is no method to distinguish tumors that will remain indolent from ones that will become aggressive. Therefore, in many cases, DCIS lesions may be overtreated [6].

For stage I–III breast cancer, localized therapies are usually implemented and include surgery followed by radiation therapy. Systemic therapies, including chemotherapeutics and other drugs (hormone suppressive therapy and Her-2 targeted therapy), are also used before (neoadjuvant) and/or after (adjuvant) surgery. Stage IV breast cancer (metastatic) is generally treated with systemic therapies, and in some cases, surgery and radiation may also be beneficial [7]. The efficacy of treatments are usually monitored using CT scans and bone scans or PET scans every 3–6 months after treatment. However, CT scans and/or PET scans present significant challenges for monitoring treatment efficacy. It can be difficult to differentiate tumor progression from pseudoprogression or infection on CT scans [8]. PET scans are expensive and are often challenging to get reimbursed by insurance providers. Many of these imaging techniques require the administration of iodinated contrast for accurate monitoring and interpretation, which can be nephrotoxic. There is a definite need for a less time-consuming and less invasive test to monitor breast cancer during treatment. Therefore, identifying alternative methods for monitoring breast cancer treatment efficacy is of significant interest. An accurate and noninvasive assay to diagnose breast cancer, monitor tumor progression, and determine treatment efficacy over time could decrease overdiagnosis/overtreatment and aid in patient decision making during the treatment process.

Previous investigations have shown the potential of volatile organic compounds (VOCs) as biomarkers of breast cancer, along with other diseases [9,10,11,12,13,14]. VOCs present a noninvasive opportunity for disease detection, as they are differentially expressed in biological samples in the presence of disease [15,16,17]. Several groups have employed solid-phase microextraction (SPME) coupled with gas chromatography-mass spectrometry (GC-MS) to profile VOCs for breast cancer biomarker discovery [16,18,19,20]. Through instrumental and chemometric analyses, VOCs have been previously shown to be biomarkers for breast cancer in urine. One study published by Silva et al. reports a human urinary VOC biosignature that can discriminate patients with breast cancer from healthy patients with 100% accuracy by using unsupervised multivariate statistical analysis on a panel of six VOCs [21]. Further investigation by the same group identified a panel of ten urinary VOCs that can differentiate breast cancer from healthy controls with more than 90% accuracy [22]. Both studies report carbonyls (ketones and aldehydes), terpenes, and sulfur-containing VOCs as potential biomarkers for breast cancer in urine. 

A study published by the current authors explored utilizing murine urinary VOCs to track mammary tumor progression for three weeks after tumor injection. Results of this study identified a unique panel of VOCs that discriminated between healthy and tumor-bearing mice with 94% accuracy and differentiated the end points (cancer week 1 and cancer week 3) with 97% accuracy. In the same study, we also reported a principal component regression model that predicts the number of days after tumor injection with R^2^ equal to 0.71, indicating the capability of VOCs to noninvasively monitor breast cancer progression in real time as early as one week after induced tumor injection [23]. Another study based on the same cohort of mice investigated the use of pitavastatin as a therapeutic agent. Statins inhibit 3-hydroxy-3-methylglutaryl-CoA (HMG-CoA), the enzyme leading to the biosynthesis of cholesterol [24]. Therefore, these drugs also inhibit the production of mevalonate and are used clinically to lower cholesterol [25]. Blocking the production of mevalonate and cholesterol has been previously demonstrated to induce antitumor properties [26,27,28]; therefore they were previously used to treat induced mammary tumors in mice. This study showed that pitavastatin administered at high doses (8 mg/kg body weight) resulted in significant antitumor effects. This was also reflected in the VOC profile analyzed three weeks after tumor injection and pitavastatin treatment [29]. However, urine samples were not analyzed in mice administered pitavastatin at low doses, and the previous studies did not profile VOCs over the course of time. Herein, we extend these previous studies using the same cohorts of mice [23,29] to analyze the ability of urinary VOCs to determine the efficacy of pitavastatin (pita) at high and low doses over the course of three weeks using two approaches (approach 1 and 2). 

## 2. Results

### 2.1. Urine Collection and Data Screening

A total of 139 urine samples were collected and aliquoted from mice across four sample classes over the course of three weeks. A total of 20 urine samples were collected before mice were injected with tumor cells, serving as the control class. A total of 45 urine samples were collected from the cancer sample class (cancer week 1 (12), cancer week 2 (15), and cancer week 3 (18)). A total of 33 urine samples were collected from the pita high (PH) sample class (PH week 1 (7), PH week 2 (12), and PH week 3 (14)), and 41 urine samples were collected from the pita low (PL) class (PL week 1 (7), PL week 2 (18), and PL week 3 (16)). Data screening showed 212 VOCs, which were qualified for analysis. A total of 74 of these VOCs were statistically significant (*p*-value < 0.05) between either cancer weeks 1–3 vs. control (44 VOCs) or cancer week 3 vs. control (60 VOCs), with 30 of the features identified with a *p*-value < 0.05 for both comparisons. These VOCs were subject to further investigation to probe for differences in mice treated with pitavastatin to determine treatment efficacy.

### 2.2. Univariate Statistical Analyses

**Figure 1:**
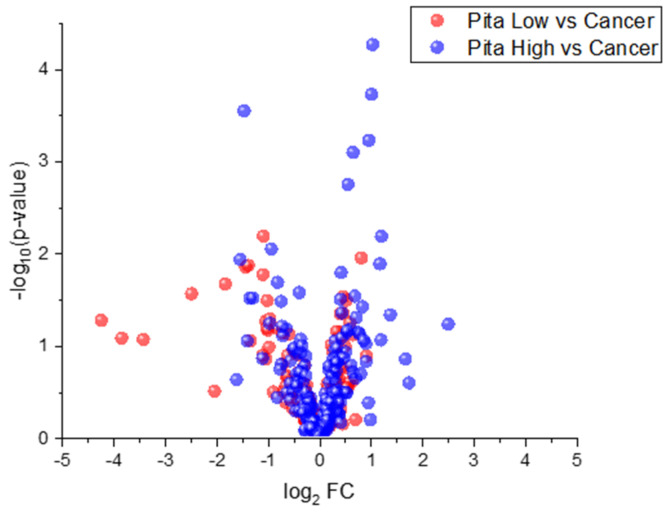
Volcano plot shows more VOCs are differentially expressed between PH (weeks 1–3) and cancer (weeks 1–3) relative to PL (weeks 1–3) and cancer (weeks 1–3).

Prior to undertaking approaches 1 and 2, PH Weeks 1–3 and PL Weeks 1–3 were compared relative to cancer weeks 1–3 using all 212 VOCs. The Student’s *t*-test was undertaken to identify statistical significance between each treatment and cancer. A volcano plot was generated by plotting the −log_10_ (*p*-value) against the log_2_ fold change (FC) for both treatments to visualize which treatment had more differences in VOC expression relative to cancer. Most VOCs were upregulated in both the PH samples and in the PL samples when compared to cancer samples. This is interesting, as the majority of VOCs were downregulated in cancer weeks 1–3 compared to control samples. There is a handful of VOCs that are downregulated in PL samples and show high absolute FC values relative to cancer; however, many of these VOCs did not display statistical significance (relatively high *p*-values). Moreover, the volcano plot demonstrates that PH presents more considerable differences in VOC expression relative to PL when compared to cancer (VOCs here have relatively lower *p*-values in PH relative to PL), which is initially indicative that PH treatment is more effective when compared to PL treatment (Figure 1).

**Figure 2:**
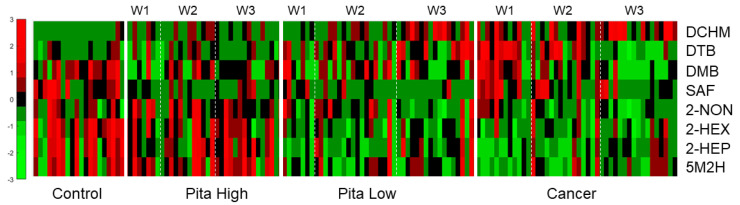
Hierarchical heatmap for the eight VOCs differentially expressed when comparing PH to cancer using approach 1. These VOCs show significant differences between cancer weeks 1–3 and control samples. PH shows a high degree of similarity with the control, and PL samples mirror VOC expression in the cancer sample class.

Approach 1 was then undertaken to screen for differences between PH and cancer in a time-dependent fashion. Upon implementation of two-tailed Student’s *t*-tests between PH week 1 vs. cancer week 1, PH week 2 vs. cancer week 2, PH week 3 vs. cancer week 3, and PH weeks 1–3 vs. cancer weeks 1–3, a total of eight VOCs (Supplementary Table S1) were statistically significant (*p*-value < 0.05) across two or more comparisons. Two VOCs (2-nonanone (2-NON) and dicyclohexylmethanone (DCHM)) were significant between PH week 3 and cancer week 3, whereas five VOCs (2-hexanone (2-HEX), 2-heptanone (2-HEP), 5-methyl-2-hexanone (5M2H), 2-NON, and DCHM) were significant for PH weeks 1–3 vs. cancer weeks 1–3 after adjusting *p*-values for false discovery rate (FDR). A hierarchical heatmap was generated using these eight VOCs (the five noted above, along with 3,3-dimethyl-2-butanone (DMB), 2,4-di-tert-butylphenol (DTB) and safranal (SAF)) (Figure 2). VOCs within cancer weeks 1–3 are mostly downregulated upon tumor injection and throughout progression (compared to control samples). Some VOCs displayed low intraclass variation across all weeks of cancer, and other features were progressively dysregulated from cancer week 1 to cancer week 3. Control samples for all VOCs presented in the heatmap have high reproducibility between replicates. The heatmap also shows that VOCs within PH weeks 1–3 appear to be expressed at similar levels as the control class, which is seen as early as the first week of treatment. On the other hand, the PL treatment sample class shows that VOCs are downregulated when compared to control samples and showed a high degree of similarity to cancer weeks 1–3 samples. There are limited differences in VOC expression between the weeks within the PH and PL treatment samples.

Approach 2 was then undertaken to screen for univariate differences in PH relative to cancer week 3 and highlighted 30 VOCs (Supplementary Table S2) differentially expressed across two or more of the following comparisons: control vs. cancer week 3, PH week 1 vs. cancer week 3, PH week 2 vs. cancer week 3, and PH week 3 vs. cancer week 3. VOCs found to be significant by FDR have an underlined asterisk in Supplementary Table S2; 3 VOCs were found to be significant between PH week 1 vs. cancer week 3, 12 VOCs between PH week 2 and cancer week 3, and 2 VOCs were significant between PH Week 3 and cancer Week 3. VOCs in Supplementary Table S2 were utilized for multivariate analysis. Overall, the univariate statistical analyses showed that VOCs had higher statistical significance between pita high and cancer samples when compared to pita low and cancer samples. In addition, the results displayed similarities in urinary VOC expression between the pita high and control sample classes, with both sample classes demonstrating differences when compared to the pita low and cancer samples.

### 2.3. Multivariate Chemometric Analyses

#### 2.3.1. Approach 1

**Figure 3:**
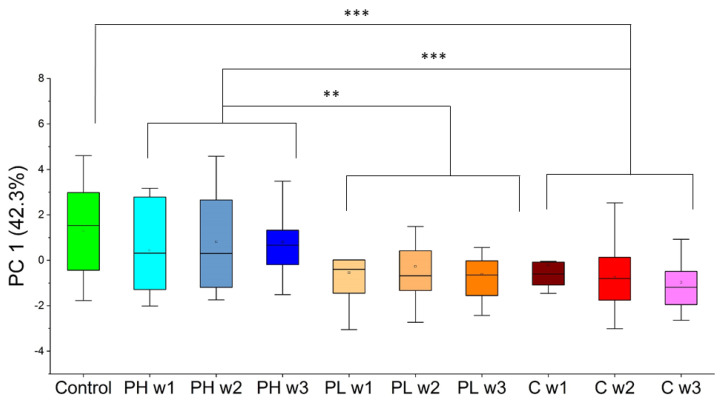
PC 1 scores in one dimension using the VOCs highlighted through approach 1 for all sample classes of interest (PH = pitavastatin high, PL = pitavastatin low, C = cancer). ** *p*-value < 0.01, *** *p*-value < 0.001.

Principal component analysis (PCA) was undertaken to visualize global data patterns using VOCs identified through both approach 1 and approach 2. Scores of the first principal component (PC 1) using the eight VOCs from approach 1 can be observed in Figure 3. PC 1 in this case accounts for 42.3% of the variance in the sample data. Significantly, PH clusters with control (*p*-value relative to Control = 0.33) and PL clusters with cancer (*p*-value relative to cancer weeks 1–3 = 0.47). Along the same lines, PL shows low *p*-values when compared to control samples (*p*-value = 0.0023). PH and control samples, on the other hand, demonstrate high statistical significance relative to cancer, with *p*-values < 0.001. Furthermore, VOCs are differentially expressed when comparing the PH treatment to the PL treatment, with a *p*-value equal to 0.0057. Differences in VOC expression are clearly seen as early as the first week of treatment, and no correlations with time were observed for this panel of VOCs when analyzed by PCA for all of the sample classes presented.

**Figure 4:**
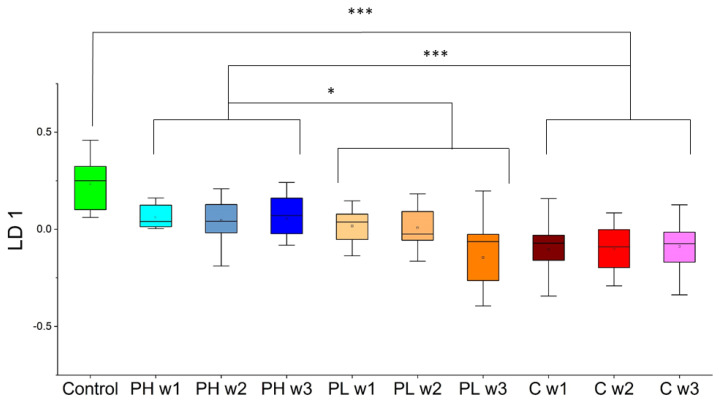
One-dimensional LDA plot where the model was trained to classify control from cancer and tested on pita high and pita low samples (PH = pitavastatin high, PL = pitavastatin low, C = cancer). * *p*-value < 0.05, *** *p*-value < 0.001.

Continuing with the VOCs highlighted from approach 1, forward feature selection coupled with linear discriminant analysis (LDA) was implemented to distinguish control and cancer week 1–3 samples. This method identified a panel of four VOCs (DCHM, 5M2H, DTB, and 2-HEX) with the ability to distinguish cancer from control with sensitivity = 91% and specificity = 100%. No significant trends over the course of three weeks were observed within the cancer samples. This model of four VOCs was then tested on the PH and PL samples. The first linear discriminant can be visualized for all sample classes (both training and testing) in Figure 4. PH samples displayed an intermediate response in between cancer weeks 1–3 and control samples in the LDA plot. PH trends towards healthy controls and showed significant differences when compared to cancer (*p*-value < 0.001) and is also statistically significantly different from PL (*p*-value = 0.015). No correlation over time was observed for the PH samples. However, PL is time-dependent, trending towards cancer and clusters with cancer week 1–3 samples by the third week (PL week 3 vs. cancer week 1–3; *p*-value = 0.42). This is observed because PL week 3 was significantly different from PL week 1 (*p*-value = 0.023). The PL samples throughout the three weeks also showed significant differences when compared to the control (*p*-value < 0.001). Multivariate analyses using approach 1 showed that both large and small panels of VOCs identified that pita low samples were trending in the direction of cancer samples, whereas pita high samples displayed a response more similar to that of the control sample class.

#### 2.3.2. Approach 2

**Figure 5:**
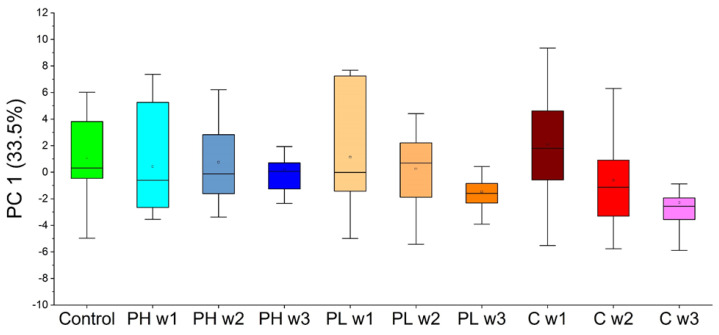
PC 1 scores in one dimension using the VOCs highlighted through approach 2 for all sample classes of interest (PH = pitavastatin high, PL = pitavastatin low, C = cancer). No significant trends are observed in the data.

For approach 2, PCA was implemented on the 30 VOCs identified as statistically significant by univariate analysis in a similar fashion to the VOCs identified in approach 1, and PC 1 accounted for 33.5% of the variance in the samples. However, these results showed high variability within sample classes, no interesting observable trends, and no statistical significance between classes of interest. All sample classes showed similar correlations over time, further indicating that this large panel of 30 VOCs is not useful for monitoring the efficacy of pitavastatin (Figure 5).

**Figure 6:**
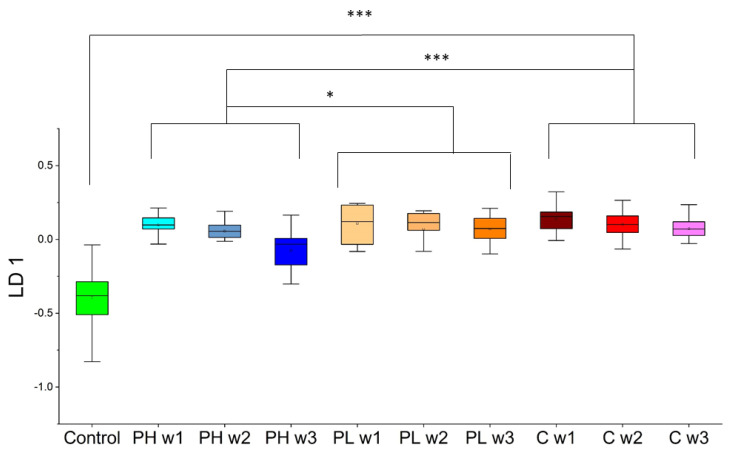
One-dimensional LDA plot where the model was trained to classify control from cancer week 3 and tested on cancer weeks 1-2, pita high and pita low samples (PH = pitavastatin high, PL = pitavastatin low, C = cancer). * *p*-value < 0.05, *** *p*-value < 0.001.

Next, although PCA did not generate any relevant trends, sample analysis proceeded with supervised multivariate statistical analysis. A supervised approach using a small panel of VOCs trained to distinguish cancer week 3 may more effectively show the impact of treatments. LDA was trained initially to distinguish cancer week 3 from control. Through LDA, a panel of five compounds (2-HEP, 2,2,4-trimethyl-1,3-pentanediol diisobutyrate (PDIB), 1,3,5-trichlorobenzene (TCB), DCHM, and 2-NON) was identified that accurately differentiated control and cancer week 3 with sensitivity = 100% and specificity = 100%. Cancer weeks 1 and 2, along with PH weeks 1–3 and PL weeks 1–3, were then tested using this panel, and the resulting LD 1 scores are shown in Figure 6.

Using this model, cancer weeks 1–2 samples showed similar trends as cancer week 3 when tested (no correlation over the course of time). On the other hand, PH week 1 was statistically significant relative to PH week 3 (*p*-value = 0.0012), trending towards control, indicating that PH can also be monitored in a time-dependent fashion using this LDA model. Additionally, in the presence of PH treatment, the panel of VOCs is differentially expressed relative to both cancer (*p*-value < 0.001) and PL (*p*-value = 0.027) across all three weeks. Lastly, the PL treatments clustered with cancer week 1–3 samples and showed large differences when compared to the control sample class (*p*-value < 0.001). Unlike the previous approach, there were no observable differences among PL weeks 1–3. Taken as a whole, unsupervised analysis through approach 2 yielded no significant differences between sample classes of interest. However, supervised LDA showed significant differences between pita low and pita high, with pita high samples trending significantly toward control samples and pita low clustering with cancer samples. 

## 3. Discussion

The volcano plot (Figure 1) shows that many more VOCs are differentially expressed in the presence of pitavastatin treatment at high concentration relative to pitavastatin administered at low concentration when both sets are compared to cancer without treatment. This aligns well with previously reported X-ray and micro-CT results, which showed that pitavastatin at high concentrations was an effective treatment, whereas when administered at low concentrations, no antitumor effect was observed [29]. Furthermore, the hierarchical heatmap (based on approach 1, Figure 2) shows similar results, where VOCs are mostly downregulated in the cancer class and in the pita low class relative to control and pita high samples. VOCs in the pita high class are expressed similarly to the healthy controls, whereas the VOCs in the pita low samples are more similar to cancer week 1–3 samples. In a previous paper, we identified VOCs of breast cancer, some of which did change over time (modeling cancer progression) and some of which did not [23]. In this case, statistical methods were not trained to identify differences due to tumor progression, and therefore, were more in favor of those VOCs remaining consistently dysregulated.

Employing PCA in approach 1 enabled the separation of sample classes of interest (Figure 3). Four VOCs (DCHM, 5M2H, DTB, and 2-HEX) were identified using LDA in approach 1 (Figure 4). Pita high samples showed significant differences relative to cancer in the direction of control samples, and pita low samples showed significant differences relative to control in the direction of cancer samples. There were also differences between pita high and pita low samples, and pita low demonstrated significant differences over time. Approach 2 was used to probe differences between each week of pita high and cancer week 3 (the endpoint). PCA was implemented on this set of VOCs, and no significant differences were observed between the samples of interest (Figure 5). LDA results using five VOCs (2-HEP, PDIB, TCB, DCHM, and 2-NON) identified through approach 2 (Figure 6) showed that pita high samples had significant differences in the direction of healthy samples (opposite direction of pita low and cancer samples). These results show that VOCs can predict the efficacy of pitavastatin treatments and mirror traditional analyses as early as the first week after tumor injection and treatment. The previous investigation on the efficacy of pitavastatin was conducted by micro-CT, X-ray analysis, mechanical testing, and histological imaging, which showed the same results as those obtained from VOCs in mouse urine, namely that pita high slows tumor progression and inhibits tumor-induced osteolytic lesions, whereas pita low does not alleviate osteolysis [29]. For an example, X-ray analysis and histological imaging showed decreased osteolytic lesions within pita high samples, whereas micro-CT images showed proximal tibia destruction due to tumors, which was alleviated upon administration of pita at high doses. Furthermore, pita high significantly increased the mechanical strength of the tibia, whereas pita low did not [29].

Interestingly, many of the VOCs have been previously reported as biomarkers for breast cancer. For example, 2-HEX and many other ketone bodies have been reported by Silva et al. [30] and in our previous studies [17,23] to be potential biomarkers of breast cancer, as they are biological products of lipid peroxidation. Ketones have also been implicated as potential biomarkers for other diseases, including hypoglycemia and different cancer types [4,31,32,33]. In our previous studies, ketone expression was downregulated in the presence of mammary tumors. However, in this study, we report that their expression is enriched when mice receive pitavastatin treatment at high concentrations, which is indicative of the treatment efficacy. A previous independent study by some of the authors of the present paper demonstrated that the upregulation of ketones is correlated with antitumor effects induced by bone loading [34]. In vitro assays demonstrated that two specific ketones (2-pentanone and 2-HEP) reduce tumor cell viability, which was also correlated with increases in aralkylamine N-acetyltransferase (AANAT) and tyrosine hydrogenase (TH). These analytes are enzymes involved in the syntheses of dopamine and melatonin, which have also previously been shown to play a role in the suppression of tumors [34]. 

Limitations of this study include that the results were based on a relatively small sample size, and there was no independent external validation performed for the multivariate analyses. Additionally, all mice were housed in the same environment and fed the same diet. Therefore, probing VOC differences due to breast cancer and antitumor therapies in humans will be more difficult, as they will present higher heterogeneity from varying diets, levels of activity, etc. Breast cancer treatments in humans may also significantly alter VOC expression, regardless of the efficacy of the treatment, which will make utilizing VOCs for monitoring treatment efficacy more difficult. Finally, there is also an array of breast cancer treatments (surgery, radiation, systemic, etc.) which may or may not be able to be monitored using VOCs in urine. Another potential downfall of this study is the fact that no healthy control mice were given pitavastatin treatments (only tumor-bearing mice). Pitavastatin alters the mevalonate pathway [29], which is a rich source of volatile terpenes (VTs). However, the LDA panels for both approaches excluded the use of VTs and included mainly ketones and aromatics. To the best of our knowledge, there is no direct correlation between ketones/aromatics and the mevalonate pathway. The origin of VOCs in this study was speculative, and VOCs were not correlated to other types of biomarkers, including but not limited to genes, proteins, other metabolites, etc. Future studies should aim to correlate VOCs to other types of biomarkers for validation and exploring the potential origin of these molecules. The long-term goal is to translate these results to women with breast cancer, potentially aiding in the decision-making processes during treatment and decreasing overdiagnosis/overtreatment. In the short-term, however, future investigations utilizing murine models to assess the efficacy of different therapeutics may analyze urinary VOCs as an alternative or complementary method to micro-CT, X-rays, and histological techniques. 

## 4. Materials and Methods

### 4.1. Instrumentation and Materials

A two-centimeter divinylbenzene/carboxen/polydimethylsiloxane (DVB/CAR/ PDMS) SPME fiber (purchased from Sigma Aldrich, St. Louis, MO, USA) was used to preconcentrate VOCs. Guanidine hydrochloride (GHCl; pH = 8.5) was purchased from Sigma Aldrich and used to denature major urinary proteins (MUPs), as they bind VOCs in hydrophobic pockets [35]. Headspace vials with a volume of 10 mL were purchased from Thermo Fisher Scientific (Waltham, MA, USA) and were used for urine sample storage and analysis. An Agilent (Santa Clara, CA, USA) 7890A GC system coupled to an Agilent 7200 MS quadrupole time-of-flight (QTOF) equipped with a PAL autosampling system (CTC Analytics, Zwingen, Switzerland) was used to incubate, extract, and analyze VOCs. The GC column utilized for VOC separation was an Agilent Ultra Inert HP-5 ms with 30 m length, 250 μm internal diameter, and 0.25 μm film thickness. MATLAB R2020a and Origin were used to generate figures. 

### 4.2. Tumor Injection, Drug Administration, and Urine Collection

All procedures conducted were approved by the Indiana University Animal Care and Use Committee and complied with the Guiding Principles in the Care and Use of Animals, supported by the American Physiological Society (APS). Twenty female BALB/c mice (6 weeks old) were acquired from Harlan Laboratories (Indianapolis, IN, USA) and injected in the iliac artery with 4T1.2 mammary tumor cells obtained from Dr. R. Anderson at the Peter MacCallum Cancer Institute (Melbourne, VIC, Australia). Pitavastatin (Livalo) was administered each day via intraperitoneal injection at high (8 mg/kg body weight) and low (4 mg/kg body weight) doses to subsets of mice after tumor injection. All tumor-bearing mice were sacrificed on day 21 after tumor injection, as this timeline allowed for sufficient tumor progression and treatment effect. Physical changes due to tumor progression and treatment effect were evaluated by measuring tumor-induced bone damage by micro-CT, X-ray analysis, mechanical testing, and histological imaging [29]. Prior to tumor injection, urine from all 20 mice was obtained to serve as the control group. Following tumor injection, urine was collected from both treated (at low and high doses) and untreated tumor-bearing mice for three weeks. Mice were caged at room temperature and fed the same diet (mouse chow ad libitum) during the experiment. Urine was collected over dry ice using Pasteur pipettes into glass centrifuge tubes. Furthermore, 50 μL aliquots of urine were transferred to a 10 mL headspace vial and stored in a −80 °C freezer before SPME GC-MS QTOF analysis was implemented.

### 4.3. SPME GC-MS QTOF Analysis

Urinary VOCs were detected and analyzed through headspace SPME coupled to GC-MS QTOF. The DVB/CAR/PDMS SPME fiber was conditioned before the first sample was run each day and between runs. Because a limited amount of urine was collected from the mice, one aliquot was analyzed per sample. GHCl was added to urine samples in a 1:1 volumetric ratio prior to GC-MS analysis. Urine samples were heated to 60 °C for 30 min prior to SPME to drive VOCs into the headspace. The preconditioned SPME fiber was then inserted into the vial and incubated in the sample headspace for 30 min to extract VOCs. Upon completion of extraction, the SPME fiber was inserted into the inlet of the GC (at 250 °C) for two minutes to thermally desorb VOCs. The chromatographic protocol began by maintaining the oven at 40 °C for two minutes, which was followed by an 8 °C/min ramp to 100 °C, a 15 °C/min ramp to 120 °C, an 8 °C/min ramp to 180 °C, a 15 °C/min ramp to 200 °C, and finally, an 8 °C/min ramp to 260 °C. The mass transfer line was held at 250 ℃ during the chromatographic run. Electron ionization at 70 eV was used for the MS with a source temperature of 250 ℃. The MS operated in full-scan mode, scanning from 26 to 400 amu with a rate equal to 5 spectra per second.

### 4.4. Data Processing and VOC Identification

Deconvolution and spectral alignment of chromatographic peaks across samples was based on mass-to-charge ratio (m/z) and retention time similarities and performed in MassHunter Profinder. Silanes and siloxanes, degradation products of both the GC column and SPME fiber, were removed from the matrix, as these are regarded as instrumental and method artifacts. Features appearing in less than 50% of either control or cancer weeks 1–3 sample classes were excluded from further analyses. Data normalization was performed through MS Total Useful Signal (MSTUS). Finally, MSTUS values were autoscaled (z-scored) to generate a matrix with a similar signal range. Upon completion of data screening, VOCs were identified through Agilent MassHunter Profinder and MassHunter Unknowns Analysis coupled with the NIST17 mass spectral library. Upon comparison of the features in Unknowns Analysis with the NIST17 library, VOCs with a match factor >70, along with similar non-polar retention index (NPRI), were tentatively identified. Experimental NPRI was determined through a previously implemented instrument-specific calibration curve [16,17].

### 4.5. Chemometric Analyses

Univariate statistical analyses were implemented via the Student’s *t*-test to identify VOCs differentially expressed between the sample classes of interest. Two approaches were undertaken to screen for VOCs that were potentially useful for monitoring the efficacy of pitavastatin treatment at high doses. For both approaches, only VOCs that were identified to have a *p*-value < 0.05 for all weeks of cancer and control or between cancer week 3 and control were included for analysis. Approach 1 screened for differences between treatment and cancer in a time-dependent fashion through four comparisons: pitavastatin at high doses (pita high, PH, 8 mg/kg body weight) week 1 vs. cancer week 1, PH week 2 vs. cancer week 2, PH week 3 vs. cancer week 3, and all PH (weeks 1–3) vs. cancer (weeks 1–3). Approach 2 screened for differences relative to cancer week 3 through four comparisons: control vs. cancer week 3, PH week 1 vs. cancer week 3, PH week 2 vs. cancer week 3, and PH week 3 vs. cancer week 3. These comparisons are useful, as tumor-bearing mice at this point in time (cancer week 3) should show the most significant differences relative to healthy controls. For both approaches, VOCs that were significant for multiple comparisons (at least two of those listed above) were identified and further utilized. Pitavastatin at low doses (pita low, PL, 4 mg/kg body weight) was not used to probe VOC differences initially, as this treatment did not show antitumor capabilities [29]. *P*-values were adjusted using the Benjamini–Hochberg procedure to account for false discovery rates [36].

Volcano plots were generated by plotting statistical significance against FC values to visualize the difference between PH vs. cancer and PL vs. cancer. Hierarchical heatmaps were generated for VOCs that were identified through approach 1. Multivariate chemometric analyses were also carried out to observe global data patterns and build classification models. PCA was applied to VOCs with a *p*-value < 0.05 across two or more comparisons for both approach 1 and approach 2 to visualize the highest amount of variation in the sample data with relatively low dimensions. Forward feature selection coupled with LDA was then applied to VOCs highlighted through approach 1 and approach 2 to build classification models and test the pitavastatin-treated samples. For the VOCs identified using approach 1, LDA was initially trained to classify cancer weeks 1–3 from healthy control samples. Samples belonging to PH weeks 1–3 and PL weeks 1–3 were independently tested using this classification model. On the other hand, for VOCs that were significant for approach 2, LDA was trained to classify cancer week 3 from control samples. Then, samples from cancer weeks 1–2, PH weeks 1–3, and PL weeks 1–3 were independently tested using this model of VOCs. Two-tailed Student’s *t*-tests were utilized to demonstrate significant differences in the multivariate output (both PCA and LDA) between the sample classes of interest (control, cancer weeks 1–3, PH weeks 1–3 and PL weeks 1–3).

## 5. Conclusions

The results of this study show that previously reported VOC biomarkers can be exploited to determine pitavastatin treatment efficacy. Through chemometric analyses, pitavastatin administered at high concentration was determined to have antitumor effects, as pita high trended towards healthy controls and displayed significant differences when compared to cancer week 1–3 samples. On the contrary, urinary VOC expression for pitavastatin administered at low concentration is highly reflective of cancer samples and showed considerable differences when compared to control samples; therefore, the VOC analyses indicated that the treatment is not effective in slowing tumor progression. The results from the VOC assays are highly reflective of the traditional analyses (micro-CT and X-ray analysis), which showed that pitavastatin administered at high doses inhibits tumor-induced bone damage, and pitavastatin at low concentrations does not. Upon validation of the results using humans with breast cancer, we found that urinary VOCs may be able to be used to accurately and noninvasively monitor the efficacy of breast cancer treatments in real time in the future, which would potentially decrease overtreatment and treatment-related morbidity without reducing survival rates.

## Data Availability

Data presented in this study is available upon request from the corresponding author.

## Supplementary Materials

The following supporting information can be downloaded at: https://www.mdpi.com/article/10.3390/molecules27134277/s1, Table S1. VOCs of interest identified using approach 1, with *p*-values when comparing each week for pita high (PH) samples and cancer (C) samples (ns—no significance, * *p* < 0.05, ** *p* < 0.01, *** *p* < 0.001, underlined asterisk—*p* < 0.05 by FDR, **name bolded**—LDA); Table S2. VOCs of interest identified using approach 2, with *p*-values when comparing each week for pita high (PH) samples to cancer (C) week 3 samples (ns—no significance, * *p* < 0.05, ** *p* < 0.01, *** *p* < 0.001, underlined asterisk—*p* < 0.05 by FDR, **name bolded**—LDA).

## Sample Availability

No biological samples or volatile organic compound standards are available from the authors.
