# Supplementary material for: Chemometric Analysis of Urinary Volatile Organic Compounds to Monitor the Efficacy of Pitavastatin Treatments on Mammary Tumor Progression over Time
Source: Molecules. 2022 Jul 3;27(13):4277. doi: 10.3390/molecules27134277 (PMC9268606; doi:10.3390/molecules27134277)
Supplement: Supplementary file 1 [file molecules-27-04277-s001.zip › molecules-1763641-supplementary.pdf]

Supplementary Information

Chemometric Analysis of Urinary Volatile Organic Compounds to Monitor the Efficacy of Pitavastatin Treatments on Mammary Tumor Progression Over Time

Paul Grocki [1,2], Mark Woollam [1,2], Luqi Wang [3,4], Shengzhi Liu [3,4], Maitri Kalra [5], Amanda P. Siegel [1,2], Bai-Yan Li [3], Hiroki Yokota [2,4,6,7,8] and Mangilal Agarwal [1,2,9,*]

1 Department of Chemistry and Chemical Biology, Indiana University-Purdue University, Indianapolis, IN 46202, USA; pgrocki@iu.edu (P.G.); mwoollam@iu.edu (M.W.); apsiegel@iupui.edu (A.S.); agarwal@iupui.edu (M.A.)

2 Integrated Nanosystems Development Institute, Indiana University-Purdue University, Indianapolis, IN 46202, USA

3 Department of Pharmacology, School of Pharmacy, Harbin Medical University, Harbin 150081, China; luqicmu160@163.com (L.W); szliu@ccmu.edu.cn (S.L.); liby@ems.hrbmu.edu.cn (B.L.)

4 Department of Biomedical Engineering, Indiana University-Purdue University, Indianapolis, IN 46202, USA; hyokota@iupui.edu (H.Y.)

5 Hematology and Oncology, Ball Memorial Hospital, Indiana University Health, Muncie, IN 47303, USA; mkalra@IUHealth.org (M.K.)

6 Simon Comprehensive Cancer Center, Indiana University School of Medicine, Indianapolis, IN 46202, USA

7 Indiana Center for Musculoskeletal Health, Indiana University School of Medicine, Indianapolis, IN 46202, USA

8 Biomechanics and Biomaterials Research Center, Indiana University-Purdue University, Indianapolis, IN 46202, USA

9 Department of Mechanical & Energy Engineering, Indiana University-Purdue University, Indianapolis, IN 46202, USA

* Correspondence: agarwal@iupui.edu

Table of Contents

Table S1: VOCs of interest identified using approach 1, with *p*-values when comparing each week for pita high (PH) samples and cancer (C) samples (ns – no significance, * $p < 0.05$, ** $p < 0.01$, *** $p < 0.001$, underlined asterisk – $p < 0.05$ by FDR, **name bolded** – LDA).

Table S2: VOCs of interest identified using approach 2, with *p*-values when comparing each week for pita high (PH) samples to cancer (C) week 3 samples (ns – no significance, * $p < 0.05$, ** $p < 0.01$, *** $p < 0.001$, underlined asterisk – $p < 0.05$ by FDR, **name bolded** – LDA).

Table S1. VOCs of interest identified using approach 1, with *p*-values when comparing each week for pita high (PH) samples and cancer (C) samples (ns – no significance, * $p < 0.05$, ** $p < 0.01$, *** $p < 0.001$, underlined asterisk – $p < 0.05$ by FDR, **name bolded** – LDA).

VOC ID	Retention Index	Abr.	PH W1 vs. C W1	PH W2 vs. C W2	PH W3 vs. C W3	All PH vs. C
**2-Hexanone**	761	2-HEX	ns	*	*	***
2-Heptanone	865	2-HEP	ns	**	*	***
**2,4-Di-tert-butylphenol**	1519	DTB	**	ns	*	*
2-Nonanone	1091	2-NON	ns	*	***	**
3,3-dimethyl-2-butanone	690	DMB	*	ns	*	ns
**Dicyclohexylmethanone**	1576	DCHM	ns	ns	***	***
Safranal	1201	SAF	*	ns	ns	*
**5-Methyl-2-hexanone**	819	5M2H	ns	ns	*	***

Table S2. VOCs of interest identified using approach 2, with p-values when comparing each week for pita high (PH) samples to cancer (C) week 3 samples (ns – no significance, * $p < 0.05$, ** $p < 0.01$, *** $p < 0.001$, underlined asterisk – $p < 0.05$ by FDR, **name bolded** – LDA).

VOC ID	Retention Index	Abr.	PH W1 vs. C W3	PH W2 vs. C W3	PH W3 vs. C W3
**Dicyclohexylmethanone**	1576	DCHM	**	**	***
**2-Nonanone**	1091	2-NON	*	**	***
2-Hexanone	761	2-HEX	ns	*	*
2,4-Di-tert-butylphenol	1519	DTB	ns	*	*
3,3-dimethyl-2-butanone	690	DMB	ns	*	*
2-Undecanone	1291	-	ns	**	**
8,8,9-Trimethyl-deca-3,5-diene-2,7-dione	1453	-	ns	*	*
2-Acetyl-5-methylthiophene	1119	-	ns	*	*
1,3,5-Undecatriene	1121	-	*	ns	*
Benzaldehyde	936	-	*	ns	*
Benzoic acid, ethyl ester	1160	-	*	*	ns
**1,3,5-Trichlorobenzene**	1220	TCB	**	***	ns
5-Isobutyl-2-isopropyl-3-methoxypyrazine	1452	-	ns	**	*
1,4-Benzenediol, 2-methoxy-	1311	-	ns	*	ns
**2-Heptanone**	865	2-HEP	ns	ns	*
**2,2,4-Trimethyl-1,3-pentanediol diisobutyrate**	1605	PDIB	ns	ns	**
10-Dodecanol	1435	-	ns	ns	*
Sesquiterpene	1419	-	**	**	ns
Propanoic acid, 2-methyl-, 2-ethyl-3-hydroxyhexyl ester	1415	-	*	**	ns
Dibutyl phthalate	1919	-	**	**	ns
2-Butanone	554	-	**	ns	ns
2-Buten-1-one, 1-(2,6,6-trimethyl-1,3-cyclohexadien-1-yl)-	1440	-	*	ns	ns
Thujaplicin	1400	-	ns	**	ns
Phenol, 4-(1,1-dimethylethyl)-2-methyl-	1341	-	*	ns	ns
3,5-di-tert-Butyl-4-hydroxybenzaldehyde	1774	-	ns	***	ns
2-Ethyl-5-n-propylphenol	1334	-	ns	**	ns
Ethoxyquin	1717	-	*	ns	ns

Toluene	769	-	ns	**	ns
Naphthalene	1179	-	ns	*	ns
Benzene, 1-methoxy-4-(1-methylpropyl)-	1217	-	ns	ns	*
